# The sugar transporter ZmSWEET11 participates in plant autophagy to respond to salt stress

**DOI:** 10.1080/21645698.2026.2640757

**Published:** 2026-03-18

**Authors:** Zhiyang Xing, Zengyuan Tian, Tongxian Chen, Haoyu Zang, Jiaying Wang, Shuting Zheng, Yuqi Guo

**Affiliations:** a School of Life Sciences, Zhengzhou University, Zhengzhou, China; b School of Agriculture and Biomanufacturing, Zhengzhou University, Zhengzhou, China

**Keywords:** Arabidopsis, AtSWEET11, maize, plant autophagy, salt stress, ZmSWEET11

## Abstract

The sugar will eventually be exported transporters (SWEET) proteins play an important role in plant growth, development and stress responses. However, current research on the function of SWEET proteins in maize in response to salt stress is limited. In this study, we characterized the function of *ZmSWEET11*, a gene involved in salt stress response and autophagy induced by salt stress in plants. *ZmSWEET11* is primarily expressed in the stems and is significantly induced by salt stress at maize seedlings stage. Subcellular localization analysis showed that ZmSWEET11 is localized to the plasma membrane. Gene silencing of *ZmSWEET11* via the technology of virus-induced gene silencing (VIGS) impaired maize salt tolerance and autophagic activity. Furthermore, we also determined that ZmSWEET11 interacts with autophagy-related (ATG) proteins (ZmATG2a, ZmATG2b, ZmATG8e and ZmATG18f). Overexpression of *ZmSWEET11* in Arabidopsis led to enhanced salt tolerance and increased autophagosome abundance, whereas the *atsweet11* mutant exhibited salt sensitivity. Taken together, our study demonstrated that ZmSWEET11 improves salt tolerance and autophagic activity both in maize and Arabidopsis seedlings, thereby mediating tolerance to abiotic stresses in plants. This is the first confirmation of a direct interaction between ZmSWEET11 and autophagic proteins, and shows that autophagy is closely with sugar transport in response to salt stress, thereby filling a gap in understanding the molecular mechanism of autophagy.

## KEY MESSAGE

Our study demonstrated that ZmSWEET11 (sugar will eventually be exported transporters) improves salt tolerance by regulating autophagic activity both in maize and Arabidopsis seedlings, thereby mediating tolerance to their abiotic tolerance under salt stress.

## Introduction

The growth and development of sessile plants are affected by continuously changing abiotic stresses throughout their life cycle. In response, plant transcription factor genes regulate downstream stressresponsive genes to activate the antioxidant system. For example, *MxNAS3* and *MxFRO6* from *Malus xiaojinensis* enhance the tolerance of transgenic *Arabidopsis thaliana* to both high and low iron stress.^1,2^ Similarly, overexpression of *MbWRKY50* from *Malus baccata* increases cold and drought tolerance in transgenic *Solanum lycopersicum* by upregulating antioxidant capacity, activating the CBF/DREB pathway, and inducing genes involved in ABA biosynthesis.^3^ Overexpression of *MbICE1* from *Malus baccata* improves drought and cold tolerance in transgenic *Arabidopsis thaliana* by enhancing antioxidant enzyme activity, reducing membrane damage, and upregulating stress-responsive genes such as *CBF* and *COR*.^4^ The R2R3MYB transcription factor *FvMYB44* and the NAC factor *FvNAC29* from *Fragaria vesca* also enhance cold tolerance in transgenic Arabidopsis by modulating the antioxidant system and upregulating downstream stressresponsive genes such as *AtRD29A* and *AtCBF*.^5,6^

Salt stress is a major abiotic factor that disrupts seed germination, growth, development, flowering, and fruiting.^7–9^ High salt concentrations limit the normal absorption of water and nutrients in plants and cause excessive accumulation of reactive oxygen species (ROS). Additionally, salt stress leads to ionic imbalance and ultimately reduces photosynthetic rates.^10–12^ In response, plants have evolved several adaptive mechanisms to saline environments, including stomatal closure to reduce water loss, compartmentalization of Na^+^ into vacuoles to protect growing organs, and the synthesis of osmolytes and antioxidant enzymes.^9,13,14^

Plant cells also actively accumulate compatible solutes such as sucrose, proline, and betaine to protect the plants in salt stress. Sucrose can lower cellular water potential, and maintain turgor pressure, thus supporting basic growth and metabolic processes. *MdSPS* (sucrose phosphate synthase) catalyzes the formation of sucrose-6-phosphate from F6P (fructose-6-phosphate) and UDPG (uridine diphosphate glucose), providing “substrates” for sucrose accumulation in fruits and determining the efficiency of sucrose synthesis.^15^ *MdWRKY126* promotes sucrose accumulation by upregulating the expression of *MdSPS* and the sucrose transporter SUT, while inhibiting the activities of sucrose-degrading enzymes (SUSY and CWINV). Meanwhile, it suppresses the functions of hexose transporters (HT and TST) to reduce hexose loss.^16^ As sugar transporters, SWEET proteins (sugar will eventually be exported transporters) are responsible for the transmembrane transport of sucrose, hexoses, and other sugars. Particularly in sink organs such as fruits, they participate in sucrose unloading from the phloem, intracellular distribution, and vacuolar transport, serving as a key transport link for sugar accumulation.

Overexpression of *AtSWEET16* in Arabidopsis has led to improved germination, enhanced nitrogen use efficiency, and increased overall growth performance.^17^ *AtSWEET17*, the homologous gene of *AtSWEET16*, is involved in fructose transport to vacuoles for storage.^18^ *AtSWEET15*, also named *AtSAG29*, is involved in plant senescence and is induced by osmotic stresses through an abscisic acid (ABA)-dependent pathway.^19^ The mutation of *AtSWEET8* (synonym *Atrpg1*) gene results in loss of pollen viability.^20^ In rice, *OsSWEET11* and *OsSWEET4c* participate in pollen development and endosperm filling, respectively.^21,22^ In maize, expression of *ZmSWEET4c*, *ZmSWEET13a* and *ZmSWEET13b* increases during germination and these genes function to nourish the embryonic axis.^23^ *CsSWEET5a* is involved in hexose transport in cucumber and can compensate for the function loss of *AtSWEET8* in *atsweet8* mutant Arabidopsis plants.^24^

In addition to their role in plant growth and development, SWEETs are also necessary for plant response to biotic and abiotic stresses. Overexpression of *AtSWEET16* and *AtSWEET17* in Arabidopsis has been shown to enhance freezing tolerance.^25^ The *atsweet15* mutant exhibits enhanced cell viability under high salinity, whereas transgenic seedling overexpressing *AtSWEET15* growth shows hypersensitivity to high salinity. This indicates *AtSWEET15* plays a negative role in salt stress response.^19^ *AtSWEET11* and *AtSWEET12* are involved in vascular development and the double mutant exhibits greater freezing tolerance than the wild-type and both single mutants.^26^ *Xanthomonas oryzae pv. oryzae (Xoo)*, the causal agent of bacterial blight in rice, hijacks host cellular machinery by activating susceptibility genes of the OsSWEET family through its endogenous transcription activator-like effectors (TALEs).^27,28^ *CsSWEET1a* and *CsSWEET17* are involved in growth regulation and freezing tolerance by influencing the partitioning of sugars between the cytoplasm and the apoplast.^29^ Altogether, SWEET family gene exhibits functional diversity.

Autophagy, which was first proposed by Christian de Duve, is a highly conserved cellular process that set up self-degradation process. It can maintain cellular homeostasis and supports stress adaptation.^30–32^ Macroautophagy, microautophagy, and mega-autophagy are three types of autophagy in plants.^33^ Macroautophagy (referred to as autophagy) is the predominant autophagy form in plants.^34^ Autophagosome with a double-membrane structure is formed, which sequesters cytoplasmic components and delivers them to the vacuole for degradation and recycling in autophagy.^35^ The molecular mechanism of autophagy is highly complex, with a series of autophagy-related proteins participating in the process. Based on their distinct functions in autophagy, ATG proteins are categorized into four groups. (1) The ATG1/ATG13 kinase complex, consisting of ATG1, ATG13, ATG11 and ATG101, participates in the induction of autophagy.^36^ (2) The phosphatidylinositol 3-kinase (PI3K) complex, composed of VPS34, VPS15 and ATG6, mediates vesicle nucleation.^37^ (3) The ATG9/2/18 transmembrane complex provides membrane source for autophagy.^38^ (4) Two ubiquitin-like conjugation systems – namely the ATG8 lipidation system and the ATG12 conjugation system – facilitate the combination of ATG8 and phosphatidylethanolamine (PE), promoting membrane extension and autophagosome closure.^39–41^

Under normal growth conditions, autophagy generally remains at a basal level to maintain cellular homeostasis.^42,43^ However, it can be quickly induced by various stresses, including nutrient starvation, high-temperature stress, salt stress, drought stress, oxidative stress, hypoxia stress and pathogen infection.^44–46^ Autophagy-deficient mutants (*atg2*, *atg5*, *atg7* and *atg9*) and selective autophagy receptor mutant *nbr1* are sensitive to salt stress.^47,48^ In rice, *osatg10b* mutant seedlings exhibit decreased salt tolerance.^49^

Osmotic or oxidative stress can lead to abnormal functional regulation of sugar transporters (e.g., DsSWEET12), thereby hindering the normal absorption and distribution of sugars in cells and triggering cellular nutrient deficiency (carbon source shortage)^50^; under nutrient deficiency conditions, the activation of plant autophagy is initiated to degrade intracellular components, recycle nutrients to maintain cellular homeostasis.^51^ SWEET proteins and autophagy form a tightly coordinated regulatory network that collectively supports plant salt tolerance responses. On one hand, SnRK1 directly initiates autophagy by inhibiting the TOR pathway and phosphorylating ATG proteins, and, on the other hand, it may indirectly affect the transport activity of SWEET proteins by regulating glucose metabolism.

Maize is significant economic crop and widely cultivated worldwide. It remains unclear whether sugar transport participates in autophagy. Here, we characterized the role of ZmSWEET11 in the plant response to salt stress and further found that ZmATG2a, ZmATG2b, ZmATG8e, and ZmATG18f interact with ZmSWEET11, respectively. Additionally, we demonstrated that salt stress induces autophagy in both maize and Arabidopsis. Our results suggest that ZmSWEET11 participates in plant autophagy to respond to salt stress.

## Material and Methods

### Accession Numbers

*ZmSWEET11*, Zm00001d031647; *ZmATG8e*, Zm00001d049405; *ZmATG2a*, Zm00001d037321; *ZmATG2b*, Zm00001d045692; *ZmATG18f*, Zm00001d043239; *ZmATG1a*, Zm00001d027415; *ZmATG9*, Zm00001d028417; *ZmATG3*, Zm00001d000178; *ZmATG7*, Zm00001d011347; *ZmATG12*, Zm00001d018259; *ZmACTIN1*, Zm00001d010159; *AtSWEET11*, At3g48740; *AtACTIN 2*, AT3G18780; *AtATG5*, At5g17290; *AtATG7*, At5g45900; *AtATG10*, At3g07525; *AtATG18a*, At3g62770; *AtATG8e*, AT2G45170.

### Bioinformatic Analysis

Amino acid sequences of *ZmSWEET11* were obtained by EnsemblPlants (https://plants.ensembl.org/index.html.) and aligned by ESPript (https://espript.ibcp.fr/ESPript/ESPript/). The physicochemical properties of ZmSWEET11, including molecular weight (MW), isoelectric point (pI), instability index, and grand average of hydropathicity (GRAVY), were analyzed using the online ExPASy ProtParam tool (https://web.expasy.org/protparam/). The gene structure prediction was assessed with GSDS (https://gsds.gao-lab.org/). The protein sequences of Arabidopsis SWEETs and *Solanum lycopersicum* SWEETs were obtained from TAIR database (https://www.arabidopsis.org.) and NCBI database (https://www.ncbi.nlm.nih.gov/), respectively. Bayesian methods were applied to construct phylogenetic tree in MEGA X. Boots trap analysis was performed with 1000 replicates and the remaining parameters were default values.

### Plant Materials and Growth Conditions

Maize inbred line B73 and *Arabidopsis thaliana* line (ecotype Columbia-0, Col-0) were germinated and grown in the greenhouse with 16 h light and 8 h dark at 22°C. Three-leaf maize seedlings were sampled and used for gene expression analysis under treatment with 200 mM NaCl solution.

The *atsweet11* (SALK_073269) mutant was obtained from the AreShare (https://www.arashare.cn/index/Product/index) and was confirmed by qRT-PCR before use.

### RNA Isolation, cDNA Synthesis and QRT-PCR Analysis

Total RNA was extracted using TRIzol reagents (Vazyme, Nanjing, China) according to the manufacturer’s instructions. First-strand cDNA was synthesized from 1 µg of total RNA, using HiScript® II Q RT SuperMix for qPCR (+gDNA wiper) from Vazyme. The coding sequence (CDS) of *ZmSWEET11* was cloned from maize stems and ligated into pGM-T (Tiangen, Beijing) for sequencing. Quantitative real-time PCR (qRT-PCR) was performed on the Bio-Rad CFX96 using the LightCycler 480 II (Roche, Switzerland) and the 2^−ΔΔCt^ method was employed to quantify relative expression levels. Triplicate biological experiments were performed on each group. The *ZmACTIN1* gene and *AtACTIN2* gene were used as the endogenous control in maize and Arabidopsis, respectively. The primer sequences utilized for analysis of transcript levels are listed in Table S1.

### Obtain the Transgenic Arabidopsis Seedlings

We generated transgenic Arabidopsis overexpressing *ZmSWEET11* in wild type and *atsweet11* mutant through the floral dip method.^52^ In brief, the CDS of *ZmSWEET11* was amplified by PCR from maize genome DNA using primers (5’- AGCTAGCTAACTCTCGATCTCCA-3’ and 5’-ACTAGCAGACGACGATGCCT-3’) and The transformation vector was constructed using GatewayR Technology with the ClonaseTM Kit (Invitrogen, USA) according to the manufacturer’s protocol. The recombinant plasmid pMDC83-*ZmSWEET11* was introduced into *Agrobacterium tumefaciens* strains GV3101 by liquid nitrogen freeze-thaw method. Transformed Arabidopsis seeds were harvested from transformed plants (T_0_) and grown on 1/2 Murashige and Skoog (MS) medium containing 20 μg/mL hygromycin. Hygromycin resistant lines were selected for PCR identification. Homozygous T_3_ progeny from the T_2_ population were selected and further verified by Western blot and salt tolerance analysis.

### Confirmation of Transgenic Arabidopsis Plant

The PCR amplification was performed using genomic DNA extracted from leaves of T3 transgenic plants and control. The primers used for PCR were pMDC83-*ZmSWEET11*-FP (5”-CACCGTCGTCGTCAGCAATGGCAGG-3‘) and pMDC83-*ZmSWEET11*-RP (5’-ACGGGAGAAGCACTGCACGC-3”). The PCR program consisted of an initial denaturation at 95°C for 5 min, followed by 35 cycles of denaturation at 94°C for 30 s, annealing at 60°C for 30 s, and extension at 72°C for 1 min, and a final extension at 72°C for 10 min. The PCR products were then analyzed by electrophoresis on a 2% agarose gel.

### Western Blot Analysis of Transgenic Arabidopsis Plant

After 10 d of cultivation in 1/2 MS, the seedlings of WT and transgenic Arabidopsis plants were transferred to nutrient solution for 10 d. Total protein of was extracted from WT and transgenic Arabidopsis seedlings with a buffer consisting of 50 mM Tris/HCl (pH 8.0), 150 mM NaCl, 1 mM EDTA, and 0.2% (w/v) Triton X-100, 4% β-mercaptoethanol, 1 mM dithiothreitol (DTT) and 1% (v/v) protease inhibitor cocktail (BBI Life Science) and then used for protein quantification with BCA protein quantitative Kit (Boster). The protein samples (200 μg amounts) were electrophoresed in 8% SDS-PAGE and the gels were transferred to nitrocellulose membranes. The membranes were blocked with TBST buffer (10 mM Tris/HCl (pH 7.5), 150 mM NaCl and 0.05% Tween-20) supplemented with 5% nonfat milk for 2 h and incubated with primary antibodies (Anti-GFP antibody, abcam, diluted at 1:200) in TBST buffer with 5% BSA overnight at 4°C. Afterward, the membranes were washed three times (10 min each) with TBST buffer and incubated with the secondary antibodies (Goat Anti-Mouse IgG H&L (HRP), abcam, dilution at 1:1000) for 2 h. After being washed three times with TBST buffer, the membranes were incubated with a chromogenic agent Enhanced HRP-DAB Chromogenic Substrate Kit (Boster).^53^

### Subcellular Localization

*ZmSWEET11* was cloned without the stop codon with primers (5’- tctcgatacaccaaatcgactctagaATGGCAGGAGGCTTCTTCTC-3’ and 5’- tcctcgcccttgctcaccatggtaccCACCGCGGCGGCGGC-3’) and inserted into Super1300 and plasmid Super1300-*ZmSWEET11* was introduced into *Agrobacterium tumefaciens* strain GV3101. Later, the Agrobacterium cultured at 28°C for 24 h was collected by centrifugation and resuspended in the infiltration buffer (10 mM MgCl_2_, 10 mM EMS, 200 mM acetosyringone) to a final OD600 of 1.0, which was infiltrated into the leaves of 4-week-old tobacco (*Nicotinana benthamiana*) plants. Finally, the infiltrated leaves were harvested and observed by an LSM 880 laser scanning microscope.^54^

### Measurement of Chlorophyll and Proline Contents

Measurement of chlorophyll content was performed using ethanol as described previously.^55^ The proline content was quantified with acid ninhydrin according to the method described by Jaemsaeng et al. ^56^ All experiments were conducted with at least three biological replicates.

### PI Staining

For PI staining, the roots of 7-d-old Arabidopsis seedlings grown on 1/2 MS vertical plates with or without 100 mM NaCl were soaked in a 10 μg/ml PI solution for 3 min.^19^ Root samples were then mounted on the slide glasses and observed using an AXR NSPARC laser scanning microscope (Nikon, Japan). Furthermore, the red fluorescent dye PI was excited by a laser line of 488 nm and the emitted light is filtered between 620 nm and 710 nm.

### Monodansyl Cadaverine (MDC) Staining

MDC staining of maize leaves was done as described.^57^ Green fluorescence signals were observed using a laser scanning confocal microscope (LSM 880, Carl Zeiss, Germany). The excitation and emission wavelengths for MDC were 405 nm and 420–485 nm, respectively. For the quantification of autophagosomes, fluorescence labeling observation combined with image quantitative analysis is mainly adopted. Monodansylcadaverine (MDC) is a hydrophobic green fluorescent dye that can bind to autophagosomal membranes, so MDC staining of plant cells and tissues is often used to monitor the occurrence of plant autophagy. The fluorescent signals are quantified using image analysis software (ImageJ), specifically by counting the number of fluorescent puncta (autophagosomes) within individual cells.

### Virus Induced Gene Silencing (VIGS)

The specific silencing sequences from CDS of *ZmSWEET11* and *ZmPDS* were amplified using primers and the PCR products were subcloned into pTRV2 by homologous recombination with a ClonExpress® II One Step Cloning Kit (Vazyme, Nanjing, China). Then, the constructs pTRV2-*ZmSWEET11* and pTRV2-*ZmPDS* were transformed into *Agrobacterium tumefaciens* strain GV3101 cells by using the freeze-thaw method, respectively. For VIGS, maize seeds were surface-sterilized with 75% ethanol for 1 min, 2.5% sodium hypochlorite for 10 min, rinsed with sterile ddH_2_O for five times and soaked in sterile ddH_2_O for 24 h at 28°C. Then the immersed seed coats were scarified with a blade before co-culture. Agrobacterium liquid containing pTRV1 and Agrobacterium liquid containing pTRV2 were cultured in LB medium to an OD600 of 0.4 and mixed 1:1 as the negative control, while Agrobacterium liquid containing pTRV1 and Agrobacterium liquid containing pTRV2-*ZmPDS* were used as the positive control. The seeds were soaked in mixed agroinfiltration liquid containing acetosyringone (19.62 mg/L), cysteine (400 mg/L), and Tween 20 (5 mL/L) for 1 h with vacuum assistance. Then the mixture was placed in a shaker and co-cultured at 28°C for 10 h. Finally, the seeds were washed with sterile water and planted into nutrition soil and grown in the growth chamber. Primers used in VIGS are listed in Table S1.

### Autophagosome Formation Assay

In order to detect the formation of autophagosome, we constructed vector *proZmATG8e*-eGFP-*ZmATG8e*. The 1669 bp length of fragment located upstream of the transcription initiation site of *ZmATG8e* was amplified by PCR and inserted sites between EcoR I and BamH I of pCAMBIA1300-eGFP to replace CaMV 35S promoter. Then a genomic fragment of *ZmATG8e* (357 bp) was inserted after eGFP by homologous recombination technology and verified by sequencing. The resulting vector *proZmATG8e*-eGFP-*ZmATG8e* was introduced into *Agrobacterium tumefaciens* (GV3101) and transformed into Arabidopsis plants through the floral dipping method.^58^ The transgenic seedlings were selected by hygromycin tolerance and autophagosome formation was detected by observation on green fluorescence with microscope.

### Yeast Two-Hybrid (Y2H) Assay

The full-length CDS of *ZmSWEET11* was amplified by PCR with specific primers (Table S1), and the resulting PCR product was inserted into the EcoR I/BamH I-digested site of the pGBKT-7 (BD) vector. Similarly, the PCR product of *ZmATG2a, ZmATGb, ZmATG8e, ZmATG18f* was ligated into pGADT7 prey vector. Then, the BD-*ZmSWEET11* and AD-*ZmATG2a/b/8e/18f* vector were co-transformed into Y2HGold yeast competent cells, respectively, following the manufacturer’s instructions (Zoman, Beijing). The transformed mixture was spread on SD/-Leu/-Trp and SD/-Leu/-Trp/-His/-Ade media to observe colony growth for estimation of the self-activation assay. The BD-*ZmSWEET11* and AD-*ZmATG2a/b/8e/18f* recombinant vector was further co-transformed into yeast competent cells to validate the interaction between ZmSWEET11 and ZmATG2a/b/8e/18f. pGBKT7-p53 (BD-53) + pGADT7-largeT (AD-T) and pGBKT7-laminc (BD-Lam) + AD-T were co-transformed Y2HGold yeast competent cells as the positive control and the negative control, respectively. All the primers used for Y2H vector constructs are listed in Tables S1.

### Interaction of ZmSWEET11 and ZmATG2a/b/8e/18f in vivo by Bimolecular Fluorescence Complementation (BIFC) in Nicotiana

To analyze the interaction of ZmSWEET11 and ZmATG2a/b/8e/18f *in vivo*, the PCR products of *ZmATG2a/b/8e/18f* and *ZmSWEET11* were fused into frame of N-terminal fragment of YFP (pXY106-nYFP) and the C-terminal fragment of the YFP (pXY104-cYFP), respectively. The pXY104-ZmSWEET11 and the pXY106-ZmATG2a/b/8e/18f (with terminator) recombinant vector were co-transformed into *Agrobacterium* (GV3101) respectively by means of Agrobacterium-mediated infiltration. These plasmids were co-expressed in *Nicotiana*. The Yellow fluorescence was visualized under a confocal laser scanning microscope (ZEISS). Primers used in BIFC are listed in Table S1.

### Statistical Analysis

Quantitative data are presented as mean ± standard error (SE) with three biological replicates per group. Statistical analysis was performed using GraphPad Prism 8.0.2 (https://www.graphpad.com/), and significant differences between the means were determined by Student’s t-test or analysis of variance (ANOVA).

## Results

### Bioinformatic Analysis of the ZmSWEET Gene Family

The *ZmSWEET11* gene is 3443 base pairs long and contains four exons and three introns, which encodes a protein comprising 310 amino acids, with a molecular weight of 33.49 kDa and an isoelectric point (pI) of 9.47. The protein is predicted to be unstable and hydrophobic (Figure S1A). Multiple sequence alignment analysis indicated that the ZmSWEET11 protein contains two MtN3/saliva domains formed by seven consecutive α-helices (Figure S1B). Phylogenetic analysis revealed that both ZmSWEET11 and AtSWEET11 belong to Clade III. They exhibited the closest homology and shared 55.48% sequence similarity (Figure S2).

### Subcellular Localization and Expression Analysis of ZmSWEET11

**Figure 1.**
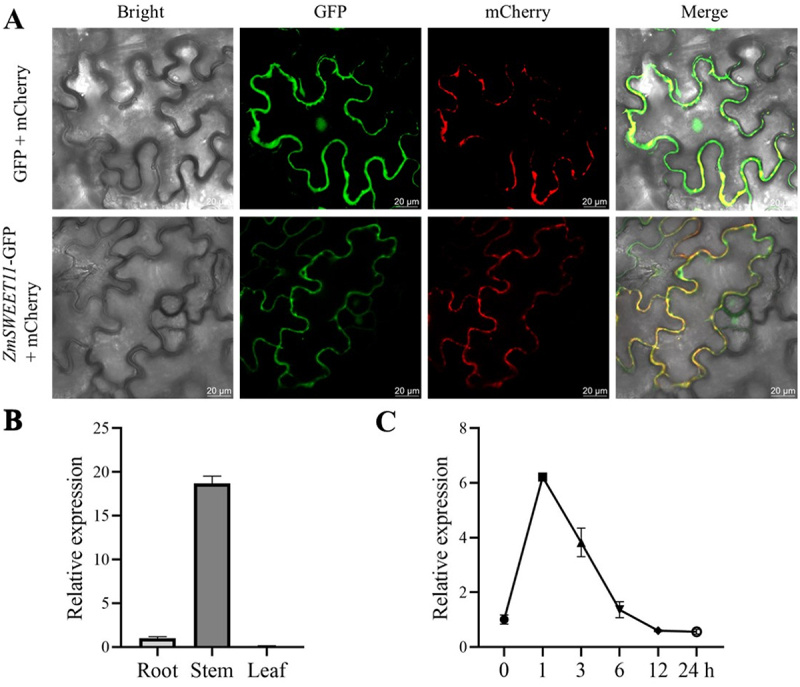
Subcellular localization and expression pattern analysis of *ZmSWEET11* gene. (A) subcellular localization of ZmSWEET11 in tobacco leaves. Membrane localization marker was used. Scale bar, 20 μm. (B) tissue specific expression analysis of *ZmSWEET11* in three-leaf maize seedlings. (C) expression analysis of *ZmSWEET11* in maize stems under salt stress. The *ZmACTIN1* gene was used as the internal reference gene. Biological triplicates were averaged. Bars represent standard error of the mean.

To analyze the subcellular localization of ZmSWEET11, we constructed a ZmSWEET11-GFP fusion vector by inserting the *ZmSWEET11* coding sequence at the upstream of GFP in the Super1300. The vector was then transiently transformed into tobacco leaves. Fluorescence observation indicated that ZmSWEET11 is localized to the plasma membrane (Figure 1A).

We then analyzed the transcription levels of *ZmSWEET11* in different tissues of maize seedlings by qRT-PCR. Under normal control conditions, the *ZmSWEET11* gene was highly expressed in stems but no expression in leaves (Figure 1B), which suggests that *ZmSWEET11* primarily functions in stems. To investigate the potential role of *ZmSWEET11* in response to salt stress, we analyzed its expression profile in the stems of maize plants subjected to 200 mM NaCl treatment. Our results showed that *ZmSWEET11* was significantly upregulated under salt stress, reaching its peak at 1 h, with expression levels approximately six times higher than those of the control (0 h) (Figure 1C).

### Overexpression of *ZmSWEET11* Enhances Salt Tolerance in Arabidopsis

**Figure 2.**
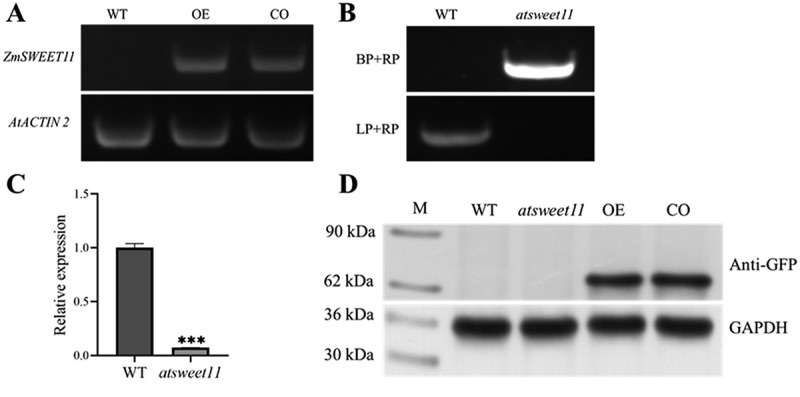
Identification of *atsweet11* mutant and overexpression of *ZmSWEET11* in *Arabidopsis* plants. (A) RT-PCR analysis of the expression levels of overexpressed *ZmSWEET11* in Arabidopsis plants. (B) PCR analysis of the T-DNA insertion in the *atsweet11* mutant. (C) qRT-PCR analysis of the expression levels of *AtSWEET11* in the *atsweet11* mutant. The *AtACTIN2* gene was used as the internal reference gene. Biological triplicates were averaged. Bars represent standard error of the mean. Significance of differences between means was statistically analyzed using student’s *t* test (***p < .001). (D) Protein expression levels of *ZmSWEET11* in transgenic Arabidopsis detected by Western blot analysis.

To investigate the function of *ZmSWEET11*, transgenic Arabidopsis plants overexpressing the *ZmSWEET11* in WT and *atsweet11* mutant were generated，which were designated as OE lines and CO lines, respectively. The homozygous T3 transgenic lines were identified (Figure 2A). The transcriptional levels of *AtSWEET11* were significantly decreased in the *atsweet11* mutant (Figure 2B,C). The protein expression levels of ZmSWEET11 in all genotypes were analyzed (Figure 2D).

We analyzed the salt tolerance of 2-week-old transgenic Arabidopsis lines on 1/2 Murashige and Skoog (MS) plates under different concentrations of NaCl. Under stress conditions, the OE lines had a better growth than WT (Figure S3A). Compared with WT, the *ZmSWEET11*-OE Arabidopsis exhibited vigorous root development (Figure S3A-B). We also examined the physiological index between the *ZmSWEET11*-OE lines and WT, including the fresh weight, root length, dry weight, the contents of chlorophyll and proline, which were significantly higher in *ZmSWEET11*-OE Arabidopsis than those of WT plants at the same NaCl concentration (Figure S3B-F). These results indicate that overexpression of *ZmSWEET11* in Arabidopsis enhances the tolerance to salt stress.

We then compared the salt tolerance between the WT, *atsweet11* mutant and *atsweet11* recovery lines. The phenotype analysis showed that a similar phenotype was found between the WT and CO lines. The root length of *atsweet11* lines was significantly inhibited under salt stress (Figure S4A, B). In addition, no significant differences in fresh weight, dry weight, chlorophyll content and proline content were found in WT and CO lines. However, these physiological parameters of *atsweet11* mutant were significantly lower than those of WT plants at the same NaCl concentration (Figure S4B-F). The above results suggest that the loss function of *AtSWEET11* in Arabidopsis decreases the salt tolerance, which also indicate that *ZmSWEET11* can compensate for the function of *AtSWEET11* in Arabidopsis.

**Figure 3.**
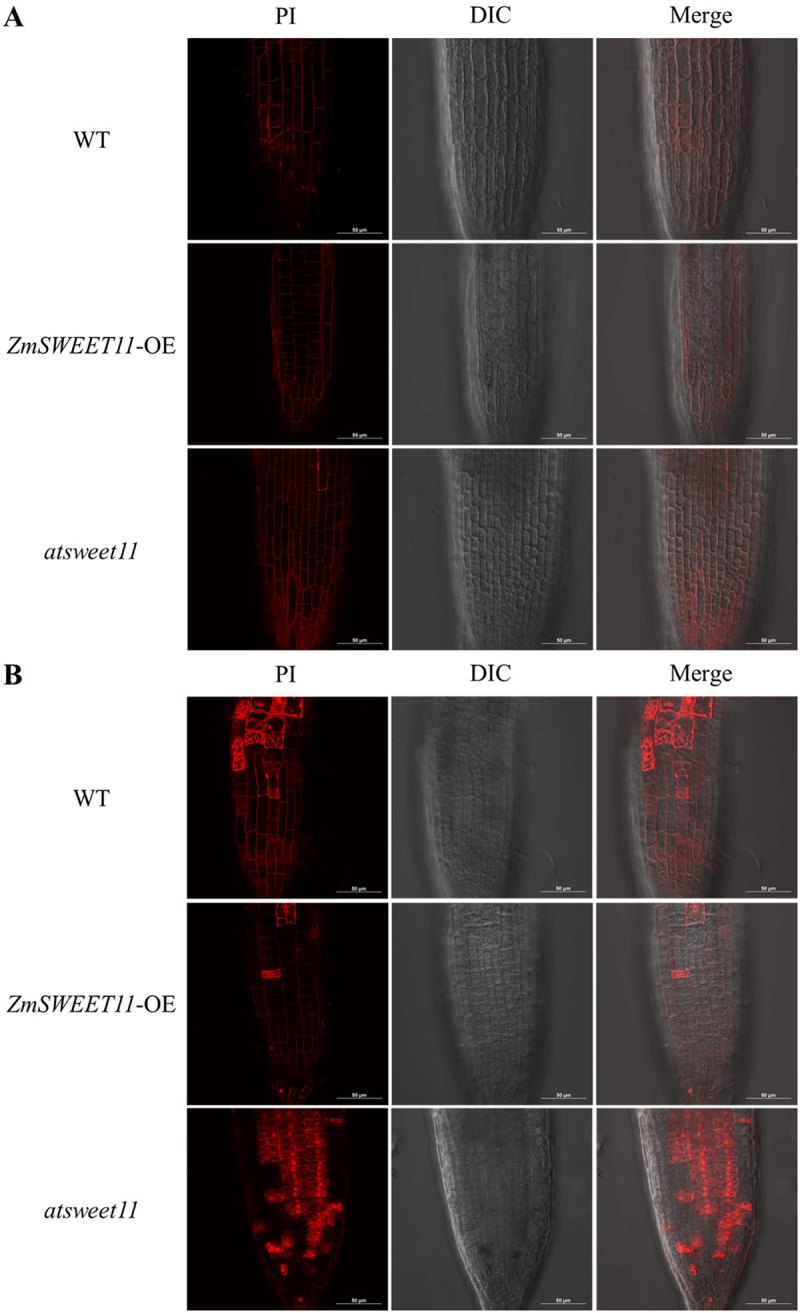
Cell viability assay. The roots of seven-day-old Arabidopsis seedlings grown on 1/2 MS vertical plates with or without 100 mM NaCl were stained with PI and analyzed by laser scanning microscope. Scale bar, 50 μm. (A) cell viability assay under normal growth conditions. (B) cell viability assay under salt stress.

The cell viability assay was performed using fluorescent dye PI that differentially stains living and dead cells. The dead cells show red fluorescence in the presence of PI, which can penetrate the plasma membrane of dead cells and bind with nucleic acids to emit red fluorescence, but it cannot penetrate the plasma membrane of living cells. Under normal conditions, no distinguishable differences were found between all the seedlings (Figure 3A). However, it is obvious that the frequency of cell death in all the seedlings increased significantly after salt treatment. The *ZmSWEET11-*OE lines exhibited lower frequency of cell death compared to the WT, as well as the *atsweet11* mutant, which indicated that the *ZmSWEET11* gene participates in cell viability regulation under salt stress (Figure 3B). Together, these results suggest that *ZmSWEET11* enhances salt tolerance in Arabidopsis.

### ZmSWEET11 Mediates Salt Stress-Induced Autophagy in Arabidopsis

**Figure 4.**
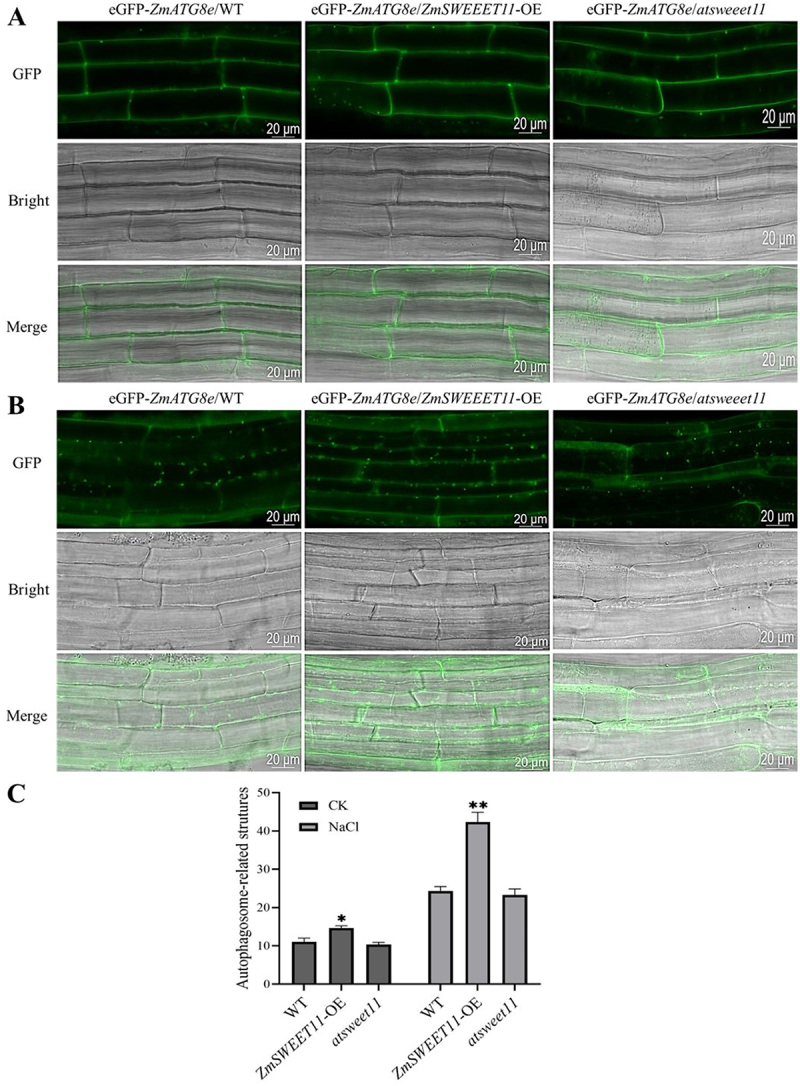
Fluorescence observation of *proZmatg8e*-eGFP-*ZmATG8e*/WT, *proZmATG8e*-eGFP-*ZmATG8e*/*ZmSWEET11*-OE and *proZmatg8e*-eGFP-*ZmATG8e*/*atsweet11* transgenic arabidopsis seedlings. (A) fluorescence observation of *proZmatg8e*-eGFP-*ZmATG8e*/WT, *proZmATG8e*-eGFP-*ZmATG8e*/*ZmSWEET11*-OE and *proZmatg8e*-eGFP-*ZmATG8e*/*atsweet11* transgenic arabidopsis seedlings under normal conditions. (B) fluorescence observation of *proZmatg8e*-eGFP-*ZmATG8e*/WT, *proZmATG8e*-eGFP-*ZmATG8e*/*ZmSWEET11*-OE and *proZmatg8e*-eGFP-*ZmATG8e*/*atsweet11* transgenic arabidopsis seedlings under salt stress. (C) quantification of green fluorescence punctate structure of transgenic arabidopsis seedlings in (A) and (B). Biological triplicates were averaged. Bars represent standard error of the mean. Significance of differences between means was statistically analyzed using student’s *t* test (*p < .05, **p < .01).

To further clarify the molecular mechanisms of *ZmSWEET11* in salt stress response, we firstly detected the transcriptional levels of some autophagy-related genes (*AtATG5*, *AtATG7*, *AtATG10*, *AtATG8e* and *AtATG18a*) in Arabidopsis. Our results showed that significant expression differences were found in the different Arabidopsis lines under salt stress (Figure S5A-E), which indicates that *ZmSWEET11* may transcriptionally regulate autophagy-related genes to enhance salt tolerance. We then observed autophagosome formation using confocal microscopy in the wild type, *ZmSWEET11*-OE and *atsweet11* lines carrying *proZmATG8e*-eGFP-*ZmATG8e* under its native promoter. One-week-old seedlings were transferred to 1/2 MS liqiud medium with or without 100 mM NaCl for 30 min, and the GFP fluorescence of root cells was subsequently observed. Under normal or stress conditions, the numbers of *proZmATG8e*-eGFP-*ZmATG8e* labeled punctate structures significantly increased in the *ZmSWEET11*-OE lines in comparison to the WT and *atsweet11* plants (Figure 4A-C). This suggests that ZmSWEET11 mediates salt stress-induced autophagy in Arabidopsis.

### Salt Stress Induces Increases in Autophagosome in Maize

**Figure 5.**
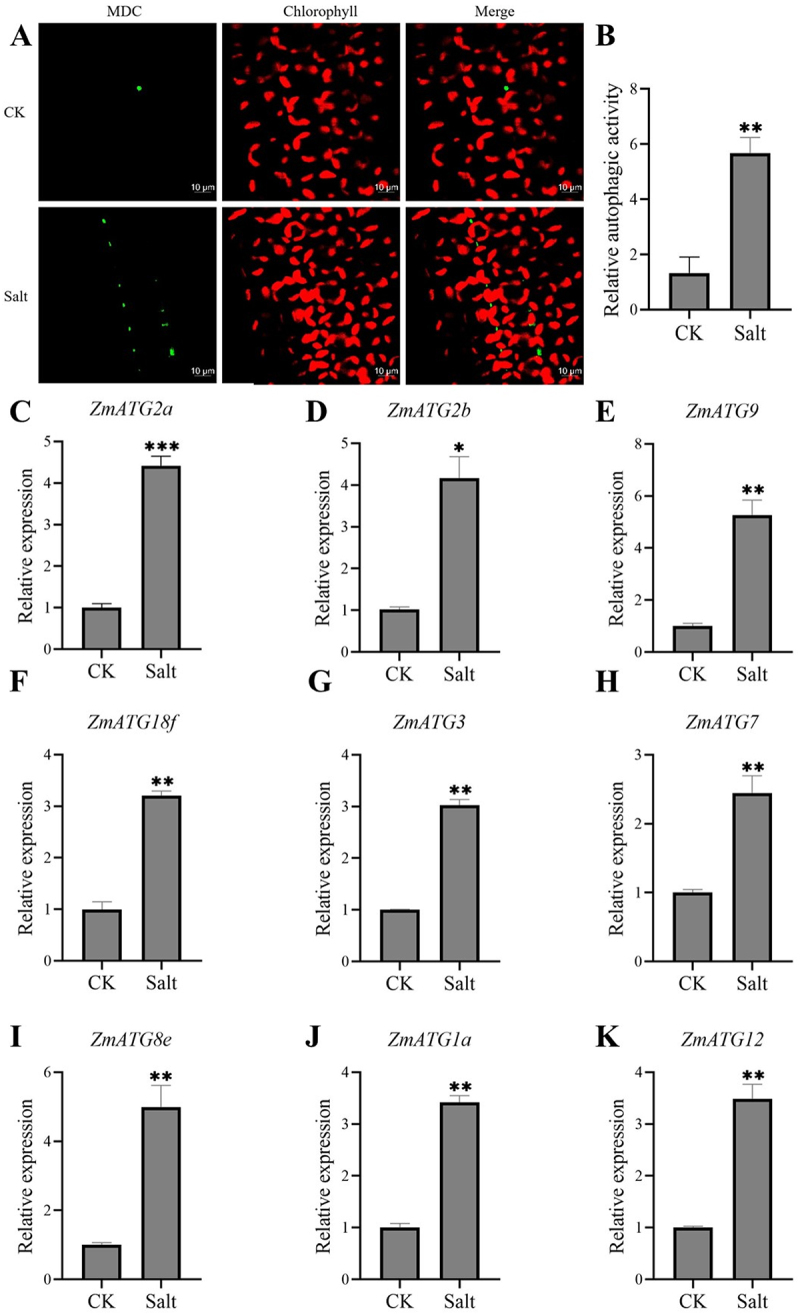
Salt stress induces autophagy in maize. (A) MDC-stained autophagosomes (green fluorescence). Maize plants at the three-leaf stage were treated with salt stress. Autophagosomes were stained by MDC in the maize leaves. Scale bars, 10 μm. (B) Relative autophagic activity under salt stress shown in (A). (C-K) qRT-PCR analysis of autophagy-related genes in maize leaves under salt stress. The *ZmACTIN1* gene was used as the internal reference gene. Biological triplicates were averaged. Bars represent standard error of the mean. Significance of differences between means was statistically analyzed using student’s t test (*p < .05, **p < .01).

To investigate whether salt stress affects autophagic flux, MDC staining was performed to detect autophagosomes formation in maize leaf cells under salt stress. As shown in Figure 5(A), the abundance of autophagosomes increased significantly in maize leaf cells subjected to salt stress compared to the control. The relative autophagic activity was 5.3-fold higher after treatment with 200 mM NaCl for 3 h than in the control (Figure 5B). Furthermore, we detected the expression levels of autophagy-related genes that are closely involved in the autophagic process using qRT-PCR under salt stress. The selected genes were induced by salt stress. Under salt stress, the expression of *ZmATG8e*, *ZmATG9*, *ZmATG2a*, *ZmATG2b*, *ZmATG18f*, *ZmATG3*, *ZmATG7*, *ZmATG1a*, and *ZmATG12* increased by 5.0-, 5.3-, 4.4-, 4.2-, 3.2-, 3.0-, 2.4-, 3.4-, and 3.5-fold, respectively. Among these, *ZmATG8e* and *ZmATG9* showed the highest induction (Figure 5C–K). These results suggest that autophagy can be induced by salt stress in maize.

### Silencing *ZmSWEET11* in Seedlings by VIGS Decreases Salt Tolerance and Reduces Salt Stress-Induced Autophagy in Maize

**Figure 6.**
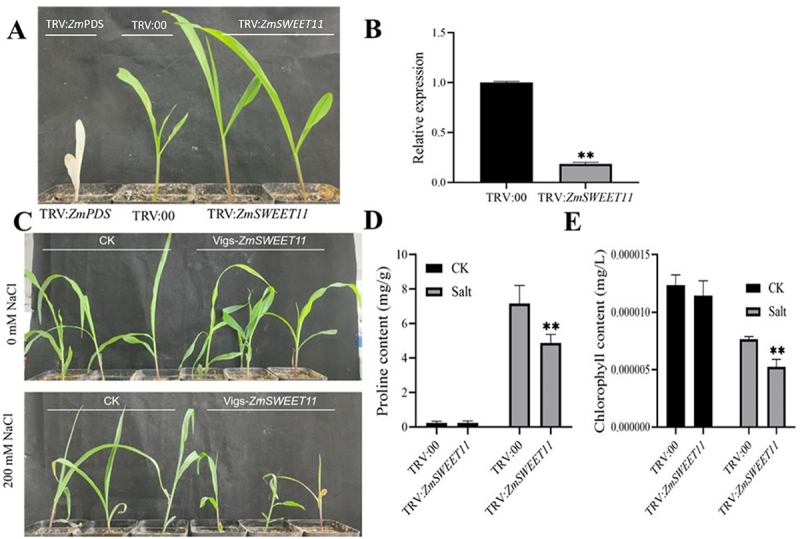
Silencing *ZmSWEET11* by VIGS decreased salt tolerance in maize. (A) Phenotypes of TRV:*ZmPDS*, TRV:00, TRV:*ZmSWEET11*. (B) Silencing efficiency of *ZmSWEET11* by VIGS. (C) Maize seedlings with *ZmSWEET11* silencing by VIGS after 200 mM NaCl treatment. (D, E) proline content (D) and chlorophyll content (E) were measured in TRV:00 and TRV:*ZmSWEET11* after treatment with 0 or 200 mM NaCl. Biological triplicates were averaged. Bars represent standard error of the mean. Significance of differences between means was statistically analyzed using student’s t test (*p < .01).

To elucidate the biological function of *ZmSWEET11* in maize, we successfully silenced *ZmSWEET11* by VIGS, which was confirmed through the positive control seedlings with photo bleaching phenotypes and significantly decreased transcriptional levels of *ZmSWEET11* (Figure 6A, B). We then compared the physiological changes between TRV:00 and TRV:*ZmSWEET11* plants under salt stress. Compared with TRV:00 plants, the silenced lines exhibited increased sensitivity to salt stress and contained lower contents of proline and chlorophyll (Figure 6C-E). These results suggest that silencing *ZmSWEET11* decreases salt tolerance in maize.

**Figure 7.**
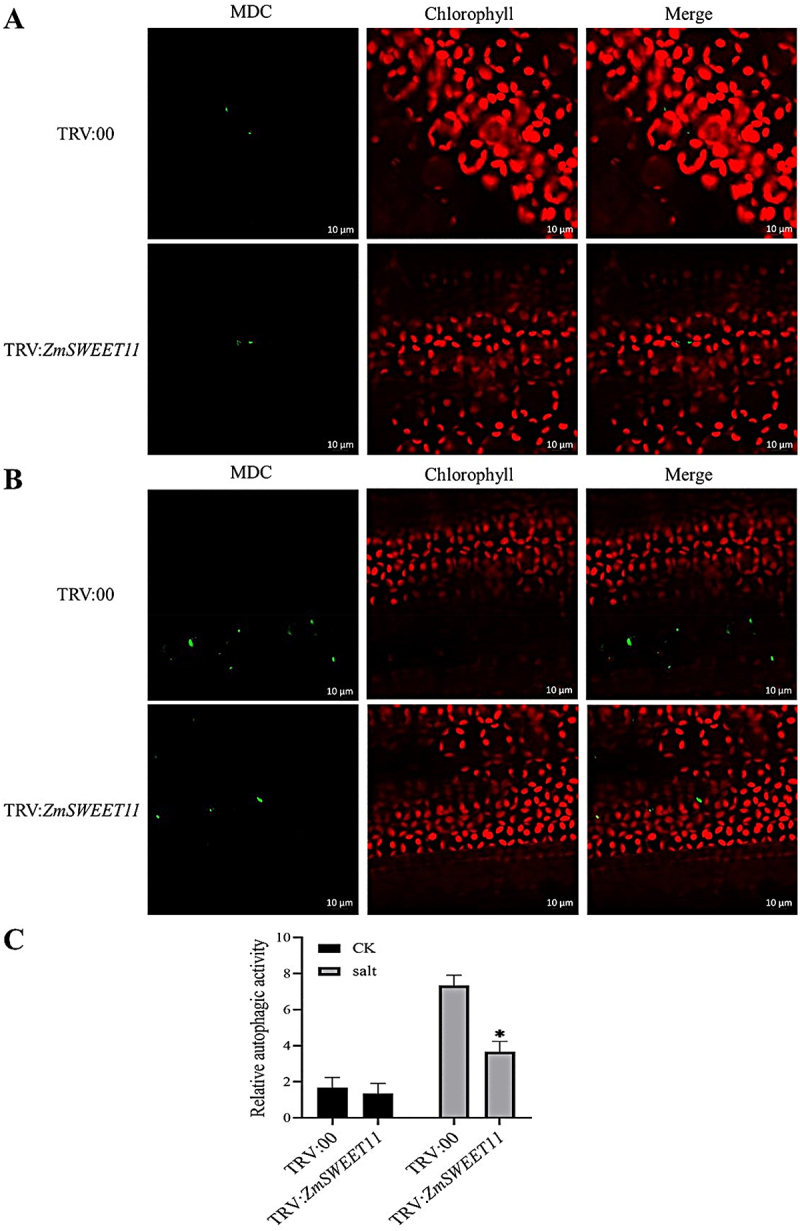
Silencing of *ZmSWEET11* gene reduced the number of autophagsome induced by salt stress in maize leaves. (A) MDC staining of TRV:*ZmSWEET11* silenced and TRV:00 control maize leaves under normal conditions. (B) MDC staining of TRV:*ZmSWEET11* silenced and TRV:00 control maize leaves under salt stress. Scale bars, 10 μm. (C) Relative autophagic activity shown in (A) and (B).

To investigate the mechanism of *ZmSWEET11* under salt stress, we observed autophagosomes in the TRV:00 and TRV:*ZmSWEET11* plants by MDC staining under salt stress. Under normal conditions, autophagosome formation showed no significant change. However, under salt stress, the number of autophagosomes was substantially higher in the vector control plants compared with the TRV:*ZmSWEET11* plants (Figure 7). We also detected the expression levels of a set of autophagy-related (ATG) genes in the TRV:00 and *ZmSWEET11*-silenced plants through qRT-PCR under salt stress. Our results showed that the expression levels of most ATG genes were significantly lower in the silenced plants compared to the control plants. This indicates that silencing *ZmSWEET11* affects the expression of genes related to autophagy (Figure S6). These findings suggest that ZmSWEET11 mediates salt stress-induced autophagy in maize.

### ZmSWEET11 Interacts with ZmATG2a, ZmATG2b, ZmATG8e and ZmATG18f

To further elucidate the mechanism of *ZmSWEET11* in maize in response to salt stress, we screened for potential ATG proteins that interact with ZmSWEET11 through bioinformatics prediction analysis. We found that ZmSWEET11 interacts with ZmATG2a, ZmATG2b, ZmATG8e, and ZmATG18f, respectively (Figure S7). In order to provide a more comprehensive understanding of the interactions between ZmSWEET11 and the ATG proteins (ZmATG2a, ZmATG2b, ZmATG8e, and ZmATG18f), molecular docking simulations were performed using Pymol software. Figures S7 display the molecular docking results of ZmSWEET11 with ZmATG2a, ZmATG2b, ZmATG8e, and ZmATG18f, respectively. These figures illustrate the potential binding sites and interaction patterns between ZmSWEET11 and each of the ATG proteins. Regions of ZmSWEET11 are shown to be in close contact with specific regions of these ATG proteins, indicating direct physical interactions. These interactions are likely to play crucial roles in the autophagy processes induced by salt stress.

**Figure 8.**
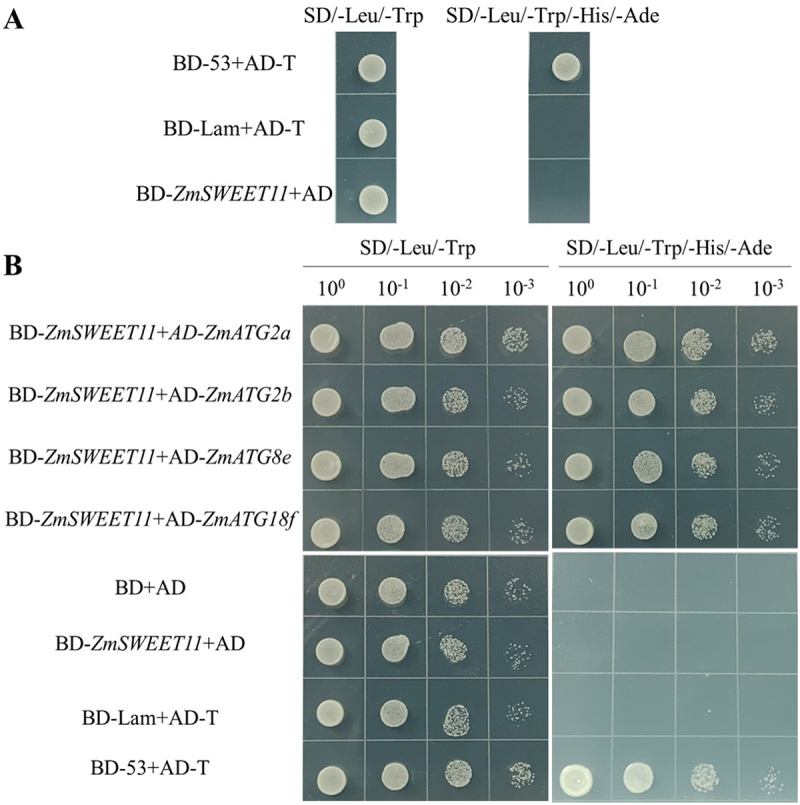
ZmSWEET11 interacts with ZmATG2a, ZmATG2b, ZmATG8e and ZmATG18f *in vivo*, respectively. (A) Self-activation detection of BD-*ZmSWEET11*. (B) Verification of the interactions between protein ZmATG2a, ZmATG2b, ZmATG8e, ZmATG18f and protein ZmSWEET11. BD-53 and AD-T were positive controls. Empty BD and empty AD, BD-Lam and AD-T, or BD-*ZmSWEET11* and empty AD were negative controls.

**Figure 9.**
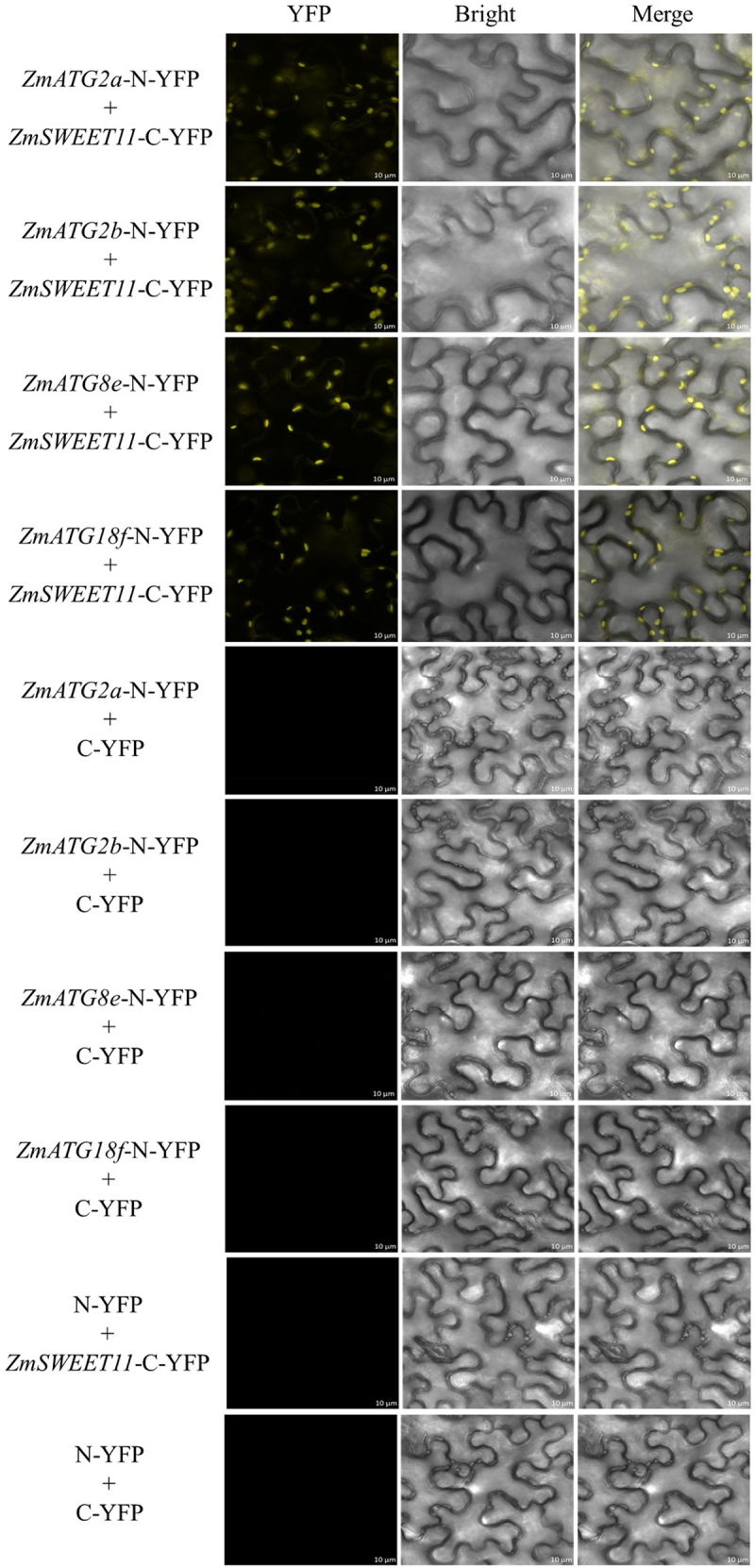
Detection of the leaf epidermal cells in tobacco by fluorescence confocal microscopy. Bar, 10 μm.

These interactions were confirmed using Y2H and BiFC assays. As shown in Figure 8A, the self-activation assay showed that pGBKT7-*ZmSWEET11* did not possess transcriptional activation activity. In yeast cells, ZmSWEET11 acted with ZmATG2a, ZmATG2b, ZmATG8e and ZmATG18f, respectively (Figure 8B). Similarly, in the BiFC assay, YFP fluorescence was observed in the leaf lower epidermal cells of *Nicotiana benthamiana* co-expressing *ZmSWEET11*-C-YFP and autophagy-related genes-N-YFP (*ZmATG2a*, *ZmATG2b*, *ZmATG8e* and *ZmATG18f*). No YFP fluorescence signals were detected in the negative control experiments (Figure 9). Taken together, these results indicate that ZmSWEET11 interacts with ZmATG2a, ZmATG2b, ZmATG8e and ZmATG18f *in vivo* and *in vitro*, respectively.

## Discussion

Beyond serving as substrates in energy production, sugars function as signaling molecules that modulate physiological processes.^58^ SWEET proteins mediate the transport and allocation of sucrose, glucose, fructose, and other sugars within plants, playing critical roles in plant development and stress response adaptation.^15^ In TRV:*ZmSWEET11* silenced plants subjected to salt stress, both sucrose and glucose levels in leaves were significantly lower than those in control plants (data not shown). The change in sucrose levels may influence the growth-defense balance of plants by regulating the expression of related genes and the activity of proteins. Under salt stress, the sucrose signaling pathway has extensive cross-talk with stress hormone signaling pathways such as abscisic acid, and jointly coordinates the stress resistance response.

There are 24 members in the SWEET transporter family of maize, which are classified into four clades, namely I-IV.^15^ The *ZmSWEET11* gene is included in clade III and its biological function in maize has not been reported yet. Our study found that salt stress induced rapid upregulation of the expression of *ZmSWEET11* within 1 h in stems of maize seedling (Figure 1C). The growth of silencing lines of *ZmSWEET11* was inhibited under salt stress (Figure 6C-E), indicating loss of *ZmSWEET11* gene decreased salt tolerance in maize. Furthermore, the *ZmSWEET11*-OE lines had a better growth condition and strong salt tolerance compared to the WT (Figure S3). *atsweet11* mutant was sensitive to salt stress, while *ZmSWEET11* can recover the phenotype of the *atsweet11* mutant (Figure S4). It is suggested that *ZmSWEET11* may be functions in the signal transduction of plant salt stress response, implying that plants will actively regulate their sugar signal pathway to counteract the damage caused by salt stress.

The previous study showed that sucrose and glucose accumulate in expanding leaves before any decline in chlorophyll content or CO_2_ assimilation rate.^59^ In our study, the AtSWEET11 mutant under salt stress exhibits impaired sugar transport and a decreased sucrose level in leaves, which ultimately results in reduced chlorophyll content (Figure S4F). These results suggest that ZmSWEET1 plays a positive role in the plant salt stress response. Changes in sucrose levels are an important signal, they modulate osmotic stress and can induce stomatal closure to reduce water loss. However, stomatal closure may also directly lead to decreased chlorophyll content and a lower photosynthetic rate. At high NaCl concentrations, sucrose can replace water molecules by interacting with proteins and membrane lipids, thereby stabilizing their native structures, preventing denaturation, and protecting membrane integrity. A mutation in AtSWEET11 may impair phloem loading and transport, hindering sucrose movement from leaves to roots and shoot tips and disrupting overall sugar redistribution, leading to sugar shortage. Consequently, limited sugar cannot be promptly allocated to young tissues, resulting in reduced growth and weakened plant resistance.

Autophagy is induced by salt stress and is essential for salt tolerance in Arabidopsis.^48^ In our study, loss of *AtSWEET11* in Arabidopsis seedlings decreased the number of autophagosomes and down-regulated the expression of autophagy-related genes under salt stress. Similarly, silenced *ZmSWEET11* caused reduction in expression of Autophagy related genes (including *ZmATG2a*, *ZmATG2b*, *ZmATG9*, *ZmATG3*, *ZmATG7*, *ZmATG8e*, *ZmATG18f* and *ZmATG12*) in maize (Figure S6). These results suggest that *ZmSWEET11* increases activity of autophagy in maize under salt stress, suggesting crosstalk between sugar signaling and autophagy that modulates cellular stress tolerance.

The core function of ATG2 is to transport phospholipids (e.g., PC, PE, PS) from the endoplasmic reticulum to the phagophore membrane via either the highly efficient “bridge” model or the less efficient “ferry” model, providing raw material for autophagosome membrane expansion.^60^ ATG8 has vital role to mediate autophagosome formation, it anchors to the autophagosomal membrane through lipidation (binding to PE), promotes phagophore elongation and maturation, and is recycled by delipidation to enable continuous autophagosome generation.^35^ ATG18 assists recycling of the autophagy protein Atg9 to the trans-Golgi network, thereby ensuring proper autophagosome formation.^61^ ZmSWEET11 interacts with ZmATG2a, ZmATG2b, ZmATG8e, and ZmATG18f (Figures S7–S8). ZmATG8e, a member of the ZmATG8 subfamily, is a ubiquitin-like protein that is conjugated to the membrane lipid phosphatidylethanolamine via a ubiquitin-like conjugation reaction; this modification is essential for autophagosome formation.^35,62^ ZmATG2 and ZmATG18 form an evolutionarily conserved complex that mediates autophagosome membrane expansion in maize: ZmATG18 stabilizes ZmATG2 at the phagophore assembly site (PAS) through direct protein–protein interaction.63 The specific interactions of ZmSWEET11 with ZmATG2a/b, ZmATG8e, and ZmATG18f coordinate autophagosome membrane formation, maturation, and targeted degradation, constituting a core molecular complex that mediates salt stress–induced autophagy and enhances plant salt tolerance.

**Figure 10.**
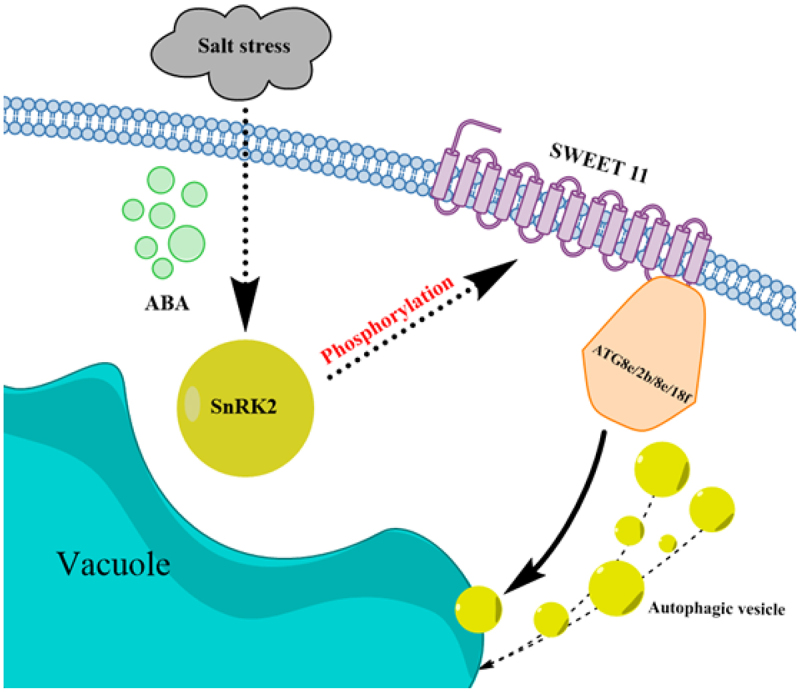
The pathway diagram of ZmSWEET11-mediated salt stress-induced autophagy.

Autophagy is a crucial intracellular recycling process linked with the TORC1 signaling network in diverse organisms. The function of TORC1 as a negative regulator of autophagy in response to biotic stress conditions is known. In optimal conditions, SnRK2s sequester SnRK1 leading to enhanced TORC1 signaling. In stress conditions, ABA signaling promotes the disassociation of the SnRK1–SnRK2 complex, and disassociated SnRKs inhibit TORC1 activity and activate autophagy process.^63^ TORC1 might be involved in the control of SnRK1 in plants through direct phosphorylation. In this study, SWEET interacts with ZmATG2a, ZmATG2b, ZmATG8e, and ZmATG18f proteins to form complex to participate in autophagy, which may promote damaged substances to degradegate.^64^ A model was established as shown in Figure 10.

In summary, under salt stress, nutrient deprivation occurs, which make plant cells may mobilize autophagy mediated by SWEET protein to degrade cellular components and macromolecules for nutrient and energy replenishment. Degraded small molecular ingredients were transported and redistributed into renewed organization to maintain the vitality of whole seedlings, which may be their survival strategy under abiotic stress.

## Supplementary Material

Supplement information.docx

## Abbreviations

ATG: autophagy-related
BiFC: bimolecular fluorescence complementation
MDC: monodansyl cadaverine; MS: Murashige and Skoog
VIGS: virus induced gene silencing
WT: wild-type
Y2H: yeast two-hybrid
CO: complementation lines.
