# Supplementary material for: The sugar transporter ZmSWEET11 participates in plant autophagy to respond to salt stress
Source: GM Crops Food. 2026 Mar 18;17(1):2640757. doi: 10.1080/21645698.2026.2640757 (PMC13003867; doi:10.1080/21645698.2026.2640757)
Supplement: Supplement information.docx [file KGMC_A_2640757_SM0886.docx]

Supplementary Table 1. Sequence information for primers used for this study

| Primers used for qRT-PCR analysis of maize ATG genes | |
| --- | --- |
| qp-*ZmATG8e*-FP | TCGAAAGGAGGCAAGCTGAG |
| qp-*ZmATG8e*-RP | CGCACCACGTACACAAACTG |
| qp-*ZmATG2a*-FP | GTGTCGGATCAGTTCTGGCT |
| qp-*ZmATG2a*-RP | GGGAGGGTCCCTGTTCCTTA |
| qp-*ZmATG2b*-FP | AGCTTGTGCTTGTCCCATCT |
| qp-*ZmATG2b*-RP | TCGGTGTTTACTAATGTTTGTGTGT |
| qp-*ZmATG18f*-FP | CTGCTGCAGCGACAGAAATC |
| qp-*ZmATG18f*-RP | TCAGAGCTTTTGGGCCCTTC |
| qp-*ZmATG1a*-FP | GTTGCCATTCACCGGGAGTA |
| qp-*ZmATG1a*-RP | ATCACGGTGCAGGAGTCTTC |
| qp-*ZmATG9*-FP | GCAGGTCTTTGCTGCTGTTT |
| qp-*ZmATG9*-RP | TGGGAGCTCCTGAGACCTTT |
| qp-*ZmATG3*-FP | GTGCCATCTCGGTTGAGGAA |
| qp-*ZmATG3*-RP | TAACATGAGCAACGGACCCC |
| qp-*ZmATG7*-FP | TTCAGTGGATGCACAAGGCA |
| qp-*ZmATG7*-RP | TAGCATCGTGGGACAACACC |
| qp-*ZmATG12*-FP | AGTTTCTTCGTCGACAACTGC |
| qp-*ZmATG12*-RP | CCCCATGCTGCCGATAAAGC |
| qp-*ZmACTIN1*-FP | CAATGGCACTGGAATGGT |
| qp-*ZmACTIN1*-RP | ATCTTCAGGCGAAACACG |
| Primers used for qRT-PCR analysis of Arabidopsis ATG genes | |
| qp-*AtATG7*-FP | GAAGATTGTCTAGGTCGTGG |
| qp-*AtATG7*-RP | CCTGCTTTCTCTTGTATCGG |
| qp-*AtATG10*-FP | ATCATACAAGGTTCCTGTGC |
| qp-*AtATG10*-RP | GATGTAGCTTGAACCATGGC |
| qp-*AtATG18a*-FP | AGATCATGCTTGCTTCGCTG |
| qp-*AtATG18a*-RP | AGAGTTCTCCGATACATCGG |
| qp-*AtATG5*-FP | ACTGATACCATGTGAAGGAG |
| qp-*AtATG5*-RP | GTATAGGCATCAAGATCACC |
| qp-*AtATG8e*-FP | AGCTGGAAGGATCAGGGAGA |
| qp-*AtATG8e*-RP | GGTCTGATGGCACAAGGTACT |
| Primers used for *proZmATG8e*-eGFP-*ZmATG8e* vector construction | |
| *proZmATG8e*-eGFP-FP | aacagctatgacatgattacgaattcTTCTAGAAACGGTCATAATCCGG |
| *proZmATG8e*-eGFP-RP | gtactagtgtcgactctagaggatccTCTTTGGATTGGATCGCTGGA |
| eGFP-*ZmATG8e*-FP | tggacgagctgtacaagtaactgcagATGGCGAGGAGCTCGTTCAA |
| eGFP-*ZmATG8e*-RP | caatgccataatactcgaacctgcagTAGCAGCCCAAAGGTGTTCTC |
| *proZmATG8e*-eGFP-*ZmATG8e*-FP | ATAGTTAACCCTTGTGGGACAC |
| *proZmATG8e*-eGFP-*ZmATG8e*-RP | AGCCTCAGCTTGCCTCCTTT |
| Primers used for Y2H vector construction | |
| BD-*ZmSWEET11*-FP | tgcatatggccatggaggccgaattcATGGCAGGAGGCTTCTTCTC |
| BD-*ZmSWEET11*-RP | tgcggccgctgcaggtcgacggatccCACCGCGGCGGCGGC |
| AD-*ZmATG2a*-FP | atatggccatggaggccagtgaattcATGGATTGGATCAATGCGATATG |
| AD-*ZmATG2a*-RP | atctgcagctcgagctcgatggatccGGGAGATGGTGTAAGGCTC |
| AD-*ZmATG2b*-FP | atatggccatggaggccagtgaattcATGGATTGGATCAATGTGATATGC |
| AD-*ZmATG2b*-RP | atctgcagctcgagctcgatggatccATCACGTCTATGCTTGAGAGG |
| AD-*ZmATG8e*-FP | atatggccatggaggccagtgaattcATGGCGAGGAGCTCGTTCAA |
| AD-*ZmATG8e*-RP | atctgcagctcgagctcgatggatccTAGCAGCCCAAAGGTGTTCTC |
| AD-*ZmATG18f*-FP | atatggccatggaggccagtgaattcATGATTTTGATATTGCAATGGCAGG |
| AD-*ZmATG18f*-RP | atctgcagctcgagctcgatggatccCAAGTCTGGGAGTTGCTCTC |
| Primers used for BIFC vector construction | |
| pXY104-*ZmSWEET11*-FP | ttacaattacaggtacccggggatccATGGCAGGAGGCTTCTTCTC |
| pXY104-*ZmSWEET11*-RP | acgctgccaccgccgtcgactctagaCACCGCGGCGGCGGC |
| pXY106-*ZmATG2a*-FP | acaacatcgaggacgccggcggatccATGGATTGGATCAATGCGATATG |
| pXY106-*ZmATG2a*-RP | cgaaagctctgcaggtcgactctagaTCAGGGAGATGGTGTAAGG |
| pXY106-*ZmATG2b*-FP | acaacatcgaggacgccggcggatccATGGATTGGATCAATGTGATATGC |
| pXY106-*ZmATG2b*-RP | cgaaagctctgcaggtcgactctagaTCAATCACGTCTATGCTTGAG |
| pXY106-*ZmATG8e*-FP | acaacatcgaggacgccggcggatccATGGCGAGGAGCTCGTTCAA |
| pXY106-*ZmATG8e*-RP | cgaaagctctgcaggtcgactctagaTCATAGCAGCCCAAAGGTGTT |
| pXY106-*ZmATG18f*-FP | acaacatcgaggacgccggcggatccATGATTTTGATATTGCAATGGCAG |
| pXY106-*ZmATG18f*-RP | cgaaagctctgcaggtcgactctagaTCACAAGTCTGGGAGTTGCT |
| Primers used for expression analysis of *ZmSWEET11* | |
| qp-*ZmSWEET11*-FP | TGCGGACTCTGGCCTACTT |
| qp-*ZmSWEET11*-RP | CTTGACCACCTTGACGATGA |
| Primers used for transgenic Arabidopsis identification | |
| qp-*AtACTIN2*-FP | CCCGCTATGTATGTCGC |
| qp-*AtACTIN2*-RP | AAGGTCAAGACGGAGGAT |
| LP | CCGAAGAGTAATGTGACCACG |
| RP | TGAAGTGGGTGCTTTTGTTTC |
| BP | ATTTTGCCGATTTCGGAAC |
| qp-*AtSWEET11*-FP | ATGTGCTTGGTTTTGCTCTC |
| qp-*AtSWEET11*-RP | TACACTTGTTCGCTTGACGA |
| Primers used for VIGS vector construction | |
| pTRV2-*ZmSWEET11*-FP | attctgtgagtaaggttaccgaattcAGGCTTCTTCTCCATGGCTC |
| pTRV2-*ZmSWEET11*-RP | agacgcgtgagctcggtaccggatccGGTCTTGACCACCTTGACGA |
| pTRV2-*ZmPDS*-FP | attctgtgagtaaggttaccgaattcGTAAAACACTTCGCACTTAG |
| pTRV2-*ZmPDS*-RP | agacgcgtgagctcggtaccggatccTTGGGACAGTTTTGTAAAC |
